# Nano-Based Biomaterials as Drug Delivery Systems Against Osteoporosis: A Systematic Review of Preclinical and Clinical Evidence

**DOI:** 10.3390/nano11020530

**Published:** 2021-02-19

**Authors:** Francesca Salamanna, Alessandro Gambardella, Deyanira Contartese, Andrea Visani, Milena Fini

**Affiliations:** Complex Structure Surgical Sciences and Technologies, IRCCS Istituto Ortopedico Rizzoli, 40136 Bologna, Italy; francesca.salamanna@ior.it (F.S.); alessandro.gambardella@ior.it (A.G.); andrea.visani@ior.it (A.V.); milena.fini@ior.it (M.F.)

**Keywords:** osteoporosis, nano-based materials, drug delivery, systematic review

## Abstract

Osteoporosis (OP) is one of the most significant causes of morbidity, particularly in post-menopausal women and older men. Despite its remarkable occurrence, the search for an effective treatment is still an open challenge. Here, we systematically reviewed the preclinical and clinical progress in the development of nano-based materials as drug delivery systems against OP, considering the effects on bone healing and regeneration, the more promising composition and manufacturing methods, and the more hopeful drugs and delivery methods. The results showed that almost all the innovative nano-based delivery systems developed in the last ten years have been assessed by preclinical investigations and are still in the preliminary/early research stages. Our search strategy retrieved only one non-randomized controlled trial (RCT) on oligosaccharide nanomedicine of alginate sodium used for degenerative lumbar diseases in OP patients. Further investigations are mandatory for assessing the clinical translation and commercial purposes of these materials. To date, the main limits for the clinical translation of nano-based materials as drug delivery systems against OP are probably due to the low reproducibility of the manufacturing processes, whose specificity and complexity relies on an adequate chemical, structural, and biomechanical characterization, as the necessary prerequisite before assessing the efficacy of a given treatment or process. Finally, an unsatisfactory drug-loading capacity, an uncontrollable release kinetic, and a low delivery efficiency also limit the clinical application.

## 1. Introduction

**Figure 1:**
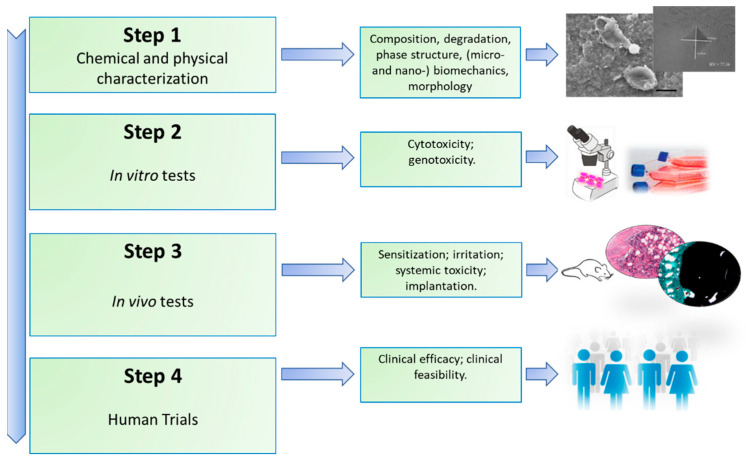
Main steps involved to establish the safety of a newly developed material.

Osteoporosis (OP) is a multifactorial disease characterized by low bone mass, altered bone quality, and enhanced skeletal fragility. It includes a complex interplay of genetic, intrinsic, and exogenous factors and lifestyles that contribute to an individual’s risk of the disease [1]. OP causes more than 8.9 million fractures annually worldwide, around 1000 per hour, of which slightly more than one third occur in Europe [1,2]. Although most prevalent in females, with one in three women over the age of 55 worldwide likely to experience an OP fracture, it is estimated that one in five men may also sustain an OP fracture after the age of 65 [2,3,4,5,6,7]. OP fractures may lead to chronic pain, disability, depression, reduced quality of life, and increased mortality; it is estimated that by 2050 the amount of hip fractures will be more than 6 million [4,5,6,7]. Considering these aspects and the continuous increase in life expectancy, OP represents a growing global health problem; thus, it is necessary to identify powerful approaches for the management of the disease. Despite the many investigations, OP treatments are not completely satisfactory and are largely restricted to anti-resorptive drugs and/or anabolic agents [8,9]. Anti-resorptive drugs—e.g., bisphosphonates, raloxifene, and denosumab—reduce the excess of bone resorption, targeting osteoclast activity [10,11,12]. The increased bone resorption can be also countered by anabolic agents, such as parathyroid hormone (PTH), growth factors, or small noncoding RNAs that are able to stimulate bone formation [13,14,15]. However, these treatments could have several drawbacks. Bisphosphonates can induce gastric side effects or fractures after prolongate use; raloxifene can cause venous thromboembolism; and denosumab can lead to hypocalcemia, anaphylaxis, or atrial fibrillation [13]. Anabolic agents, such as siRNA, might be degraded by the organism microenvironment [13]. Thus, the optimization of the use of these drugs for OP treatment—i.e., their concentrations and delivery—should be a real advancement in the field. In this context, nano-based materials seem to represent an ideal innovative platform [16]. This is probably due to nanoparticles’ (NPs) similarity in size with the architecture of the osseous tissue, since inorganic minerals and organic matrices are assembled at the nanoscale [17,18]. Moreover, NPs can take advantage of their high surface area to volume ratio, which favors the adsorption and bioactivity of neighboring proteins and cells [19]. Thus, bioactive NPs hold considerable potential in stimulating bone growth to counterbalance the increased turnover rate found during OP [19]. These properties of nano-based materials can be employed separately or together for OP treatment, particularly in the field of drug delivery and bone tissue regeneration. NPs could stabilize the bioactive agents through encapsulation or surface attachment, thus endorsing molecule internalization, targeting their delivery by cells, and permitting the control of the release of biological factors at the planned target [20,21]. Nano-based materials could also be stimulus-sensitive delivery vehicles for active substances, both chemical and biological, which lead to triggered delivery as a consequence of an external stimulus [20,21,22]. Despite the fact that using nano-based materials for the delivery of therapeutics seems to be a promising approach for OP treatment, the clinical translation of these materials is currently still far away. The safety issues with NPs have required the transfer of a large amount of knowledge from the field of pure research to that of applied research, whereas measurements techniques typical of the nanotechnological field are employed to elucidate the chemical, physical, and biomechanical properties of the nanomaterial before assessing its biocompatibility and toxicity [23]. Clinical trials following legal and ethical considerations would be also mandatory according to ISO (International Organization for Standardization) standards, as summarized in Figure 1 [24,25].

In this systematic review, we analyzed and discussed the preclinical and clinical progress in the development of nano-based materials as drug delivery systems in OP. The advantages, disadvantages, and underlying effects of nano-based materials for bone healing and regeneration in the OP condition were evaluated. Lastly, we tried to identify the most promising compositions and manufacturing methods of current nano-based materials used for OP applications, as well as the more hopeful drugs, genetic materials, or biological factors able to be delivered through these materials in OP condition.

## 2. Materials and Methods

### 2.1. Eligibility Criteria

The PICOS (Population, Interventions, Comparisons, Outcomes, Study) model was used to formulate the questions for this study: (1) studies that consider (employ) OP animals and patients, or cells derived from both (population); (2) studies that evaluate nano-based materials as drug delivery systems against OP (interventions); (3) studies that have control interventions (comparisons); (4) studies reporting the effects of nano-based materials as drug delivery systems for bone healing and regeneration in OP condition (outcomes); and (5) preclinical (in vitro and in vivo) and clinical studies (study design) [26,27]. The focus of the question was: ‘What are the main effects of nano-based materials as drug delivery in OP conditions?’ Studies from 24 April 2010 to 24 April 2020 were included in this review if they met the PICOS criteria.

We excluded studies investigating (1) nano-based materials as drug delivery systems in pathological conditions different from OP; (2) nano-based materials as drug delivery system in pathological conditions where OP is a bone manifestation of another disease (i.e., diabetes, Gaucher disease, cancer, rheumatic diseases, etc.); (3) the synthesis and characterization of nano-based materials as drug delivery systems without an associated preclinical and/or clinical study; (4) nano-based materials in which a drug delivery system was not present. Additionally, we excluded case reports, abstracts, editorials, letters, comments to the editor, reviews, meta-analyses, book chapters, and articles not written in English.

### 2.2. Information Sources and Search Strategies

Our literature review involved a systematic search conducted on 30 December 2020. We performed our review according to the Preferred Reporting Items for Systematic Reviews and Meta-Analyses (PRISMA) statement [28]. The search was carried out on four databases, PubMed, Scopus, Web of Science Core Collection, and Cochrane Central Register of Controlled Trials, to identify preclinical and clinical studies on nano-based materials as drug delivery systems in OP condition. The search was conducted combining the terms “Nano-based” AND “Materials” AND “Drug delivery system” AND “Osteoporosis”; for each of these terms, free words and a controlled vocabulary specific to each bibliographic database were combined using the operator “OR”. In addition, reference lists of relevant studies were searched for other potentially appropriate publications.

### 2.3. Studies Selection and Data Extraction

Possible relevant articles were screened using a title and abstract by two reviewers (FS and DC), and articles that did not meet the inclusion criteria were excluded. After screening the title and abstract, articles were submitted to a public reference manager (Mendeley Desktop version 1.17.9, Mendeley Ltd., London, UK) to eliminate duplicates. Subsequently, the remaining full-text articles were retrieved and examined by three reviewers (FS, DC, and AG). Any disagreement was resolved through discussion until a consensus was reached or with the involvement of a fourth reviewer (MF).

Data from the retrieved studies were tabulated taking into consideration preclinical in vitro and in vivo studies that evaluated nano-based materials as drug delivery systems in OP and clinical studies on the same topic. We extracted the following data from the preclinical studies: reference, aim, study design, experimental groups, main characteristics of nano-based material, main results. The extracted data for the clinical studies were the reference, aim, study (trial) type, patient groups (analyzed patients and number), main characteristics of the nano-based material and drug delivery strategy, quantitative measurements, and main results.

## 3. Results

### 3.1. Studies Selection and Characteristics

**Figure 2:**
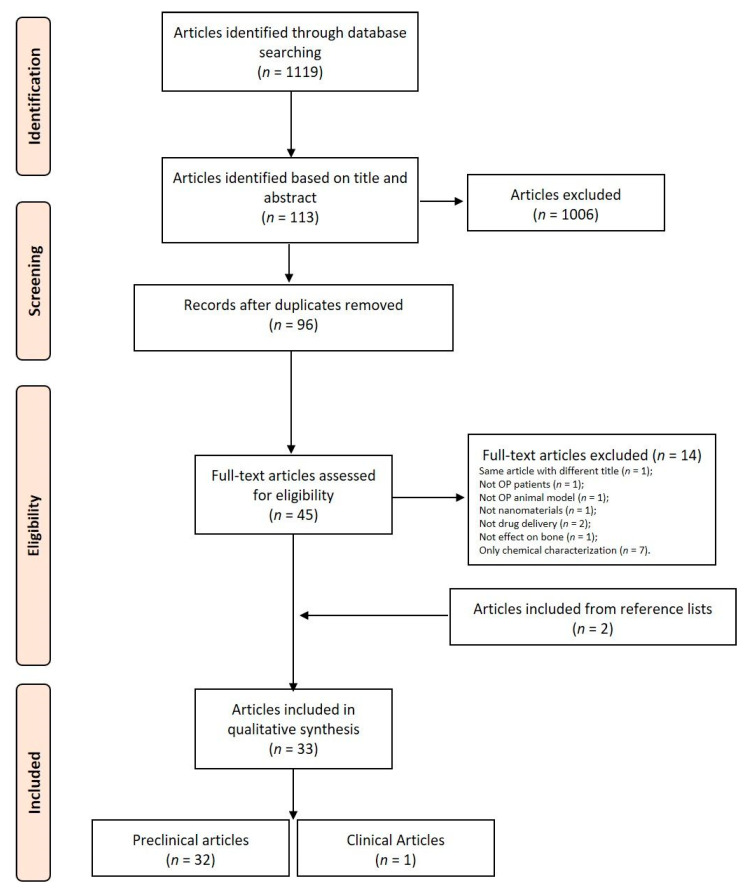
Preferred Reporting Items for Systematic Reviews and Meta-Analyses (PRISMA) flowchart for the selection of studies.

The initial literature search retrieved 1119 studies. Among those, 121 were identified using PubMed, 138 using Scopus, 99 with the Web of Science Core Collection, and 761 using Cochrane Central Register of Controlled Clinical Trials. After screening the title and abstract, 113 articles were run through the Mendeley Desktop version 1.17.9 (Mendeley Ltd., London, UK) citation manager to eliminate duplicates. The resulting 96 complete articles were then reviewed to establish whether the publication met the inclusion criteria, and 31 were considered eligible for this review. From the reference lists of the selected articles, two additional publications were found. Of the 33 articles eligible for the review, one was a non-randomized clinical study [29] while the remaining 32 were preclinical studies, of which only one used in vitro data [30,31,32,33,34,35,36,37,38,39,40,41,42,43,44,45,46,47,48,49,50,51,52,53,54,55,56,57,58,59,60,61]. The search strategy and study inclusion and exclusion criteria are detailed in Figure 2.

### 3.2. Approaches for Chemical, Physical and Structural Characterization of Nano-Based Materials

**Figure 3:**
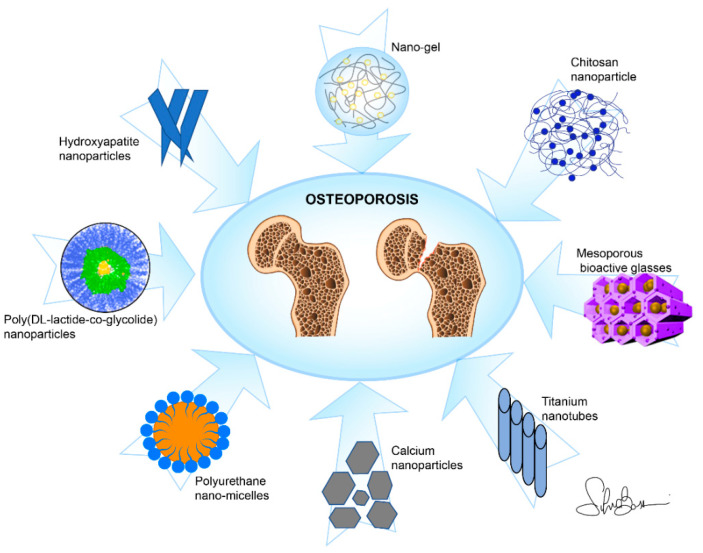
Main nano-based materials used as drug delivery systems against osteoporosis in this review.

NPs and nanocomposites (NCs) are by far the materials that were most frequently employed for drug delivery among the studies examined in this review; these also include scaffolds, nanotubes, and coatings (Figure 3). Mostly, in-liquid techniques are used for syntheses; nevertheless, in this review we highlighted some relevant characteristics of the several drugs species which are effectively delivered for the purpose of OP treatment and the methods of chemical/physical characterization specific to the NPs/NCs and aimed at determining their purity, morphology, and size.

#### 3.2.1. Hydroxyapatite (HA)-Based

HA-NPs (or nHA) alone were seldom used [35], as they were more often employed as carriers of different drugs, thus adding specific properties. Among these, bisphosphonates such as zoledronate (zoledronic acid) [41,42,48,61] and risedronate [50] were incorporated into HA-NPs. Moreover, zinc (Zn) [43] or strontium (Sr) were incorporated for invigorating bone growth and mineralization. Additionally, silver (Ag) was employed due to its well-known antibacterial activity and, at the same time, acceptable cytotoxicity [35,61]. The partial substitution of Ca^2+^ with cobalt ions (Co^2+^) and the subsequent addition of a finite magnetic moment to the otherwise diamagnetic HA molecule was also carried out, in that the Co-HA-NPs were hypothesized to favor the osteogenesis process [36]. Europium (Eu) was also incorporated into HA-NPs for the purpose of diagnostics via fluorescent imaging [40]. All the functions listed above provoked a change in the crystallinity of HA which can be visualized by X-Ray Diffraction Spectroscopy (XRD) [35,36,40,42,43,44,45,50,61]. The Fourier Transform Infrared Analysis (FT-IR) of absorption bands is also used to detect the corresponding changes in composition [35,42,43,44,45,50,61], while characteristic changes in the main dimensions and morphology of the NPs/NCs were typically monitored by Transmission Electron Microscopy (TEM) [35,39,61] and Scanning Electron Microscopy (SEM) [39,40,41,42,43,44,50]. For colloidal suspensions, chitosan [35] and calcitonin [45] were incorporated into HA-NPs and Z-potential was also used to check their stability behavior [35,61].

#### 3.2.2. Polymer-Based

*N*-(2-Hydroxypropyl)methacrylamide NPs loaded with Asp^8^-(STR-R8)-Sema4d siRNA [58] and poly(dl-lactide-co-glycolide) (PLGA) NPs loaded with 17 β-estradiol, as a drug used for hormone replacement therapy [53,54], and with risedronate sodium [34] have been proposed as novel nano-based materials for the treatment of OP. Additionally, polyurethane (PU) nano-micelles were used to deliver mRNA [31,52]. TEM was mainly used to assess the NPs morphology and size [31,34,52], eventually accompanied by Z-potential characterization [52] or differential scanning calorimetry (DSC) [34].

#### 3.2.3. Calcium-Based

Enriched milk from two types calcium (calcium citrate and calcium carbonate) was homogenized to NPs [33]. TEM was used to assess NPs morphology and structure.

#### 3.2.4. Other Nanocomposites

Functional hyaluronan-alendronate NPs embedded into a gelatine/chitosan multilayer on Ti_6_Al_7_Nb-based implants was characterized and set up to enhance the early osseointegration between the implant and the OP bone [51]. Additionally, alginate sodium, an antioxidant and anti-inflammatory bisphosphonate prepared with ampicillin in the form of NPs, and a risedronate functionalized chitosan NPs prepared by the ionic gelation technique [49] were characterized and tested, respectively, in OP patients and animals [29]. Other nanocomposites used as drug delivery systems against OP were circinal-icaritin synthesized in the form of nano-micelles [37]; nobiletine (a flavonoid with recognized anti-inflammatory activity) -loaded poly(ethylene glycol)-block-poly(e-caprolactone) nano-micelles [55]; simvastatine loaded into mesoporous HA [56]. Furthermore, the anti-OP efficacy of three-component conjugates of a succinyl spacer, a pharmacophore of 17β-amino-11α-hydroxyl-androst-1,4-diene-3-one, and a targeting sequence of RGD-tetrapeptide, was also evaluated [38]. SEM, TEM, electrospray ionization (ESI) mass spectroscopy, Atomic Force Microscopy (AFM), FTIR, Dynamic Light Scattering (DLS), and Z-potential were used to assess the obtained NPs’ and nano-micelles’ morphology and assembly.

#### 3.2.5. Scaffolds

MicroRNAs were incapsulated into biodegradable microspheres to enable a controlled delivery whose spatial control was realized by attaching the microspheres to nanofibrous polymer scaffolds [59]. Nanogel scaffolds containing mesoporous bioactive glasses were loaded with Sr, with a body temperature-controlled release [60]. These studies used SEM to image cell proliferation on the scaffold surface. Recently, porous titanium (Ti) scaffolds were loaded with zoledronic acid (ZOL) NPs [57]. All these studies, except that of Zhang et al. [60], which focused mainly on cell response, used TEM for imaging.

#### 3.2.6. Nanotubes

Nanotube arrays fabricated by electrochemical anodization were used for the controlled release of Sr/Ag [32]; Sr and Ag (the latter for antibacterial purposes) were loaded into Ti nanotubes using in-solution methods, while controlled drug release was achieved by varying the nanotube diameter. The main characterization methods specific to the imaging of the obtained structures employed SEM and/or TEM for morphological characterization as well as spectroscopic methods, such as FT-IR or ultraviolet visible spectroscopy (UV-vis) [32].

#### 3.2.7. Coatings

Sr-functionalized Ti surfaces (Sr-Ti-O) were synthesized by a physical vapor-based technique—i.e., magnetron sputtering. Depositions were carried out on grade 4 Ti implants. The coating morphology and thickness were evaluated by SEM, while XPS was used to analyze the Sr surface content [47]. Calcium-phosphate and alendronate sodium-calcium-phosphate coatings were deposited by electrostatic spray deposition on the surface of commercially pure and grit-blasted Ti implants [30].

### 3.3. Approaches for Drugs Delivery through Nano-Based Materials

**Figure 4:**
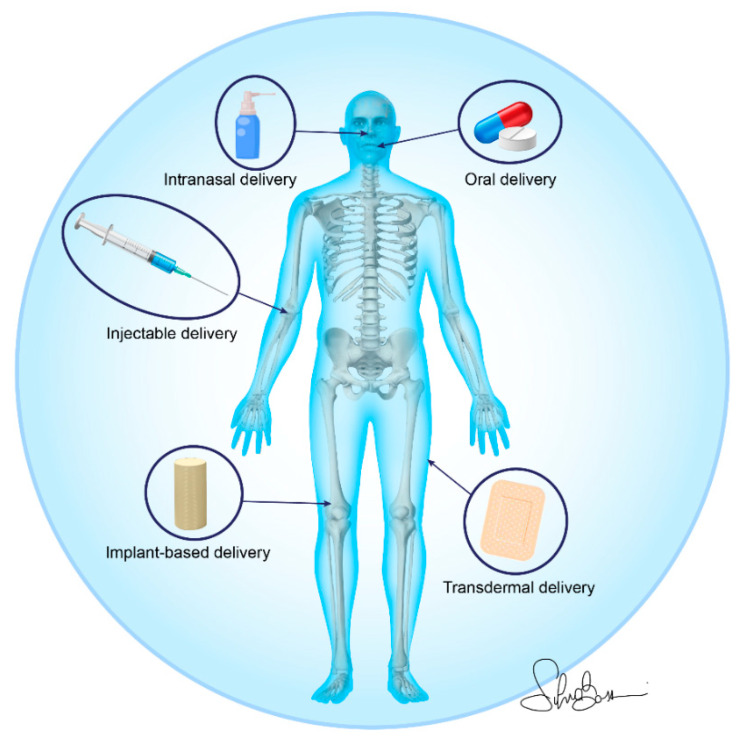
Nano-based material administration strategies for osteoporotic bone drug delivery.

In this review, the approaches to deliver drugs for OP treatment through nano-based materials include, in the preclinical in vivo studies, injectable delivery (39%), implant-based delivery (35%), oral delivery (13%), transdermal delivery (10%), and intranasal delivery (3%) [30,31,32,33,34,35,36,37,38,39,40,41,42,43,44,45,46,47,48,49,50,51,52,53,54,55,56,57,58,59,60], while the approach used in the only clinical study found in this review was oral delivery [29] (Figure 4).

The injectable delivery strategies employed in the preclinical in vivo studies comprised intravenous injection [31,35,42,43,44,50,52,58], intraosseous injection [39,41], intramuscular injection [49], and intraperitoneal injection [55,56]. All the approaches were performed using minimally invasive injection methods. The injectable nano-based materials for drug delivery—i.e., PU nano-micelles, polymers composed of organic units joined by carbamate (urethane) links [31,52], chitosan NPs [49], poly(ethylene glycol)-block-poly(e-caprolactone) (PEG-PCL) nano-micelles [55], *N*-(2-hydroxypropyl)methacrylamide NPs [58], and HA-NPs [35,39,41,42,43,44,50]—were loaded, modified, or functionalized to deliver specific anti-OP drugs. These drugs comprise, but are not limited to, polysaccharides [35], ions (Ag, Eu, Zn, Sr) [35,39,43,44], bisphosphonates (zoledronate, risedronate) [41,42,43,44,49,50], glycosaminoglycans (hyaluronic acid) [41], and flavonoids (nobiletin) [55]. In addition to these ‘traditional’ treatment options, two papers also used gene therapy strategies in order to deliver exogenous small nucleic acids—i.e., anti-miR214 [31,52].

Another simple and non-invasive route for drug administration by nano-based materials was oral delivery. However, of the 31 analyzed studies, only 4 of them employed this approach, and this could probably be due to some issues commonly linked to this strategy—i.e., drug enzymatic degradation in the gastrointestinal tract and the limited permeation across the mucosal layer [33,37,38,45]. Despite these drawbacks, several authors have developed and evaluated specific nano-based materials, such as nano-sized calcium citrate [33], nano-sized calcium carbonate [33], and HA-NPs [45], to deliver, via the oral route, skimmed milk powder enriched with vitamins B6, K1, and D3 [33]; 17β-amino-11α-hydroxyandrost-1,4-diene-3-one [38]; circinal–icaritin and suet oil self-assembled into nano-micelles [37]; and salmon calcitonin [45]. Even if few preclinical studies have used the oral delivery strategy, it is important to underline that the only clinical study included in this review used this approach for the administration of pluronic NPs and oligosaccharide nanomedicine of alginate sodium in OP patients subjected to posterior lumbar intervertebral fusion [29].

One more alternative administration route that can deliver therapeutic agents for a long period of time is represented by transdermal administration. Three authors used this administration route [40,53,54] to evaluate a nano-emulsion gel loaded with lovastatin [40] and an estradiol-loaded PLGA NPs [53,54], this last also employing iontophoresis in an attempt to increase drug permeability [54].

Finally, an additional route for the non-invasive systemic administration of drugs is the nasal pathway. Only one paper by Fazil et al. delivered a polymeric nanoparticulate formulation of sodium risedronate intranasally, showing several advantages, including the shorter time to onset of effect and the higher bioavailability due to the avoidance of hepatic first-pass metabolism [34].

Despite the minimally invasive delivery approaches above described, the implant-based delivery strategy is one of the most-used techniques in this review. Ti implants coated with alendronate-loaded calcium phosphate NPs [30], functionalized with Sr [47], loaded with Sr and Ag [32], loaded with hyaluronan-alendronate/bone morphogenetic protein-2 (BMP-2) NPs embedded into Gel/Chi multilayers [51], and integrated with ZOL loaded gelatin NPs [57] were implanted in femoral [30,51,57] and tibial defects [32,47] of mice, rats, and rabbits. In addition, paramagnetic cobalt-substituted HA-NPs [36], calcium sulfate/HA nano-cement as a carrier of BMP-2, ZOL, and bone marrow mesenchymal stromal cells (BMSCs) -derived exosomes [48], and poly (*N*-isopropylacrylamide) brush-modified mesoporous HA loaded with simvastatin [56] were implanted in femoral [48,56] and alveolar bone defects [36]. The implant-based delivery method was also used to implant a hyperbranched polymer vector for miR-26a delivery immobilized on a nanofibrous poly(l-lactic acid) (PLLA) scaffold [59] and a polymeric nanogel containing mesoporous bioactive glass loaded with Sr [60], respectively, in a mice calvaria defect [59] and in a rat femoral defect [60]. Finally, Luo et al., using the same delivery strategy, also evaluated the heterotopic bone formation of a nano-sized Sr-substituted apatite/polylactide loaded with rhBMP-2 [46] that was implanted intramuscularly.

### 3.4. Anti-Osteoporotic Effects of Nano-Based Materials as Drug Delivery System

#### 3.4.1. Preclinical Studies Results

The anti-OP effects of nano-based materials as drug delivery systems in preclinical in vivo studies are summarized in Table 1, Table 2 and Table 3. Table 1 reports the in vivo studies where the injectable delivery approach was used for the delivery of drugs through nano-based materials; Table 2 reports the in vivo studies where an implant-based delivery approach was used to deliver drugs through nano-based materials; finally, Table 3 reports the in vivo studies where oral delivery, transdermal delivery, and intranasal delivery strategies were used to deliver drugs through nano-based materials. Of the 32 preclinical studies found in this review, only one was solely in vitro, while all the others were in vivo or both in vitro and in vivo. Except for one study [30] that used an orchiectomized (ORX) animal model to induce OP, all the other studies used female animals in which OP was induced by ovariectomy (OVX) and/or by corticosteroid injection.

The only in vitro study [61] retrieved evaluated the effects of a local administration of HA-ZOL composite crystals coated with AgNPs on human OP osteoclasts co-cultured with human osteoblast-like cells. This study highlighted the influence of HA-ZOL on bone metabolism, both as a direct action on osteoclast viability and as an indirect influence on osteoclast differentiation [61]. The positive effect of ZOL on bone metabolism was further underlined by several in vivo studies where this bisphosphonate was loaded on HA-NPs (or nHA) [42] and on nHA integrated in hyaluronic acid hydrogel (nHA–ZOL–Gel) [41] and injected, respectively, in the femoral condyle and intravenously. These studies, through morphometrical analyses, sensitive biochemical markers of bone formation, and resorption and biomechanical bone strength testing revealed that the developed drug formulations were highly effective in promoting bone formation in OP animal models [41,42]. ZOL proved its efficacy also when loaded on a gelatin NP-integrated porous Ti scaffold and implanted in a rabbit femoral defect [57]. As well as alone, ZOL was loaded also in association with BMP-2 and BMSCs derived from exosomes (EXO) on a calcium sulfate/nHA-based nano-cement (NC) to enhance bone formation and healing in a femur neck canal defect in OP rats [48]. Despite the fact that in this study all treatment groups (NC-ZOL, NC-BMP-ZOL, and NC-EXO-ZOL) showed enhanced bone formation with the complete healing of the defect, an enhanced peak of fracture force was observed in NC-BMP-ZOL in comparison to all the other groups, emphasizing a synergic effect of BMP-2 and ZOL when delivered by NC [48]. In addition to the use of ZOL, some studies also used other bisphosphonates—i.e., alendronate [30,51] and risedronate [34,43,49,50]—that were functionalized, coated, or loaded on calcium phosphate NPs, PLGA NPs, nHA, chitosan NPs, and hyaluronan NPs [30,34,43,49,50,51]. As for the studies where ZOL was used, but also for those where alendronate and risedronate were used, an improved bone microarchitecture and metabolism in the presence of nano-based materials were observed independently from the delivery strategy employed. Notwithstanding the fact that ZOL and all the other bisphosphonates are potent anti-OP drugs, it is known that they did not promote bone formation or replenish the already resorbed bone. Thus, since Sr-substituted HA (SrHA) has been seen to promote bone formation and to inhibit bone resorption, Khajuria et al. investigated the effect of a SrHA/ZOL NPs injected intravenously in an OP animal model [44]. Significant improvements in bone microarchitecture, mechanical strength, serum bone-specific alkaline phosphatase, and tartrate-resistant acid phosphatase were detected when SrHA/ZOL was used [44]. Considering the key role of Sr in bone metabolism, numerous studies have evaluated its effect and that of other ions (i.e., Ag, cobalt) when loaded on different nano-based structures, such as nanotubular structures on Ti surfaces, nHA, and p(*N*-isopropylacrylamide-co-butyl methylacrylate) nanogel [32,35,36,46,47,60]. Except for one study that intravenously delivered nHA, chitosan/HA nanocomposites (nCh/HA), and Ag/HA-NPs (nAg/HA) [35], in all the other studies the nano-based materials loaded or functionalized with ions were implanted intramuscularly or in bone [32,36,46,47,60]. In the study where nHA, nCh/HA, and nAg/HA were used, superior results were seen when nHA was used alone—i.e., without chitosan or Ag [35]. In contrast, in all the other studies paramagnetic cobalt (Co)-substituted nHA mixed with autologous blood implanted in an alveolar bone defect [36], nano-sized Sr-substituted apatite/polylactide loaded with rhBMP-2 implanted intramuscularly [46], nano-topographic implants with a Sr-functionalized Ti coating (Ti–Sr–O) implanted in the tibia [47], and p(*N*-isopropylacrylamide-co-butyl methylacrylate) nanogel containing mesoporous bioactive glass loaded with Sr on BMSCs implanted in femoral defects [60] showed increased bone formation, healing, and mineralization. In addition to ions, two studies also evaluated the anti-OP effect of a transdermal nano-emulsion (NE) gel loaded with lovastatin (LNG) and of a poly (*N*-isopropylacrylamide) brush-modified mesoporous HA loaded with simvastatin (SIM) (MHA-SIM-P) both in vitro and in vivo [40,56]. The results showed the enhanced osteogenic ability of BMSCs and an improved bone microarchitecture, structure, and strength in OP rats when statins were present [40,56]. Another drug that improves bone microarchitecture and bone mineral density (BMD) is 17 β-estradiol (E2), which was trans-dermally tested once loaded on PLGA NPs also when associated with iontophoresis [53,54]. In addition to these drugs, the use of polypeptide and tetrapeptide, such as salmon calcitonin-loaded nHA [45] and the combination of an anti-OP androgen, 17β-amino-11α-hydroxyandrost-1,4-diene-3-one, RGD-tetrapeptide sequences, and a succinyl spacer in a nano-globe delivery structure [38], orally administered, turned out to be promising delivery system for OP therapy, allowing an improvement not only in BMD and bone microarchitecture but also in bone strength [38,45]. Similar results were also obtained from intraperitoneally injecting nobiletin (NOB), a polymethoxyflavone-loaded PEG-PCL (NOB-PEG-PCL) [55]. Comparable effects on bone structure and microstructure were obtained by Jiang et al., who orally administered a circinal–icaritin (CIT) suet oil (SO) self-assembled into nano-micelles under the action of sodium deoxycholate [37]. Kaur et al. also evaluated different doses of nHA (25, 50, and 100 μg/kg intravenous single dose) and a single dose of micro-sized HA (100 μg/kg) particles doped with Eu, the most reactive lanthanide that, as other ions, may substitute the calcium ion of HA [39]. By intrafemorally injecting Eu-doped nHA, a continuous improvement in ultimate stiffness and Young’s modulus of the femur shafts of rats with increased doses of nHA—i.e., from 25 to 100 μg/kg—was observed [39]. Differently, Erfanian et al. developed and evaluated two preparations of enriched milk homogenized to a nano-sized particle distribution (nano-sized enriched milks) administered by gavage in OP rats. This study showed that a nano-sized calcium carbonate-enriched-milk was more effective in preventing bone loss and fracture induced by OP than nanosized calcium citrate-enriched-milk [33].

Finally, since it is known that gene therapy is a new and alternative strategy able to regulate gene expression to treat disease by delivering exogenous small nucleic acids, such as siRNA or miRNA, several studies in this review employed this approach. Two different studies used PU nano-micelles as a delivery system for anti-miR214, employing it as the guide for delivering the miRNA drug, Asp^8^ (Asp–Ser–Ser)6 and SDSSD (Ser-Asp-Ser-Ser-Asp) peptides [31,52]. In an OVX mice model, they injected via tail vein these nano-based materials, showing an improvement in the BMD and bone microarchitecture in animals treated with Asp^8^-PU-anti-miR214 and SDSSD-PU-anti-miR214 in comparison to animals treated, respectively, with Asp^8^-PU and SDSSD-PU [31,52]. Asp^8^ was also used to set up a specific bone-targeting drug delivery system from polymeric NPs, including the incorporation of an interference molecule for Sema4d by siRNA (Asp^8^-(STRR8)-Sema4d siRNA), which was injected intravenously in OVX mice as a prevention strategy for alveolar bone loss [58]. Asp^8^-(STRR8)-Sema4d siRNA highlighted an improvement in alveolar bone structure and microarchitecture in comparison to animals treated with estrogen replacement therapy [58]. In the context of miRNAs, it is important to emphasize that one of the most widely studied polymers for DNA delivery is polyethylenimine (PEI). Zhang et al. [59] evaluated the anti-OP effect of hyperbranched polymer (HP) polyplexes (PEI and PEG) loaded with miR-26a; encapsulated in PLGA microspheres (MS); immobilized on nanofibrous (NF) PLLA scaffolds, MSCs, and osteoblasts in a calvaria defect of OVX mice. An in vitro study showed an increased expression of mineralization markers in cell-free PLLA scaffolds with immobilized PLGA 64-K MS loaded with HP/miR-26a, while the in vivo study also highlighted an improvement in BMD and bone microstructure in the same group [59].

#### 3.4.2. Clinical Study Results

The only clinical study on nano-based materials as drug delivery systems for OP patients is reported in Table 4 [29]. The study evaluated 96 OP patients treated for degenerative lumbar disease that received posterior lumbar intervertebral fusion with cages and that were treated with oligosaccharide nanomedicine of alginate sodium (ONAS) and with pluronic nanoparticles (PG) as an orally administrated control. After 1 month of therapy, yjr infection rates and side effects were lower in ONAS than those in PG, while the fusion rates were higher in ONAS than in PG. The Japanese Orthopedic Association score, used to evaluate the functional recovery of lumbar vertebrae, was higher in ONAS than in PG. The serum levels of miR-155, an miRNA involved with inflammatory responses by mediating several genes; aspartate aminotransaminase; alanine aminotransferase; and IL-1β were lower, while superoxide dismutase, glutathione, and IL-1 receptor antagonist were higher in ONAS than in PG. Thus, ONAS improves the fusion rate and reduces complications in comparison to PG and provides a better option for degenerative lumbar disease therapy.

## 4. Discussion

The interest in the use of nano-based materials as drug delivery systems is transforming the traditional drug delivery strategies used in orthopedic disorders. Here, we examined the preclinical and clinical advancements in the development of nano-based materials as drug delivery systems for OP, considering their advantages, disadvantages, and underlying effects for bone healing and regeneration. The more promising composition and manufacturing methods as well as the more hopeful drugs able to be delivered through these materials were also considered.

The nano-based materials employed as drug delivery systems for OP treatment comprise a spectrum of organic and inorganic materials that were fabricated with a plethora of techniques. Frequently, materials were combined to take advantage of the superimposition of different compositional and structural properties—such as, for example, when several drugs were delivered with HA that displayed comparable characteristics to the bone components. In-liquid techniques are the most used for syntheses; they have the main advantage of being low-cost with respect to different manufacturing methods. To obtain nano-based materials, various drugs, and several administration strategies of these drugs, are emerging for OP treatment, giving different cues for a future personalized clinical approach. Without doubt, in this review Sr as well as zoledronate and risedronate seemed to be the most used drugs delivered by nano-based materials. However, despite these ‘traditional’ treatment strategies, in this review an advanced approach has been identified in the use of specific siRNA that was employed to silence genes post-transcriptionally. Although the delivery of siRNA can offer a key tool to treat OP, these techniques are still subject to numerous questions and few preclinical studies on their delivery by nano-based materials are present. In contrast, the high usage of Sr, zoledronate, and risedronate closely reflects the clinical *scenario*, where numerous studies have been conducted to evaluate the effects of Sr due to the development of the anti-OP drug Sr ranelate and bisphosphonates for OP. Sr is able to promote osteogenic bone formation and inhibit osteoclastic bone resorption, and several clinical studies have demonstrated that Sr ranelate treatment reduces the risk of vertebral, nonvertebral, and hip fractures in OP women [62,63]. Despite these results, Sr ranelate is registered for use in Europe, but is not approved by the US Food and Drug Administration (FDA). Differently, all daily oral and one intravenous bisphosphonate (zoledronate) formulations obtained FDA approval for postmenopausal OP treatment. As for Sr ranelate, both zoledronate and risedronate have proven efficacy in bone loss prevention and fractures reduction in postmenopausal women and men with OP [64,65]. However, as most of the active drugs/substances used for the treatment of OP, Sr ranelate, zoledronate, and risedronate also possess some adverse effects, such as cardiovascular events, venous thromboembolism, myocardial infarction, gastrointestinal discomfort, and dermatitis, and, in rare cases, allergic reactions, hypocalcemia, and muscle pain [66,67,68,69,70]. Additionally, two rare (estimated at <1 case per 10,000 users) but more serious adverse effects have also been observed with bisphosphonates—i.e., atypical femoral fractures and osteonecrosis of the jaw [70]. Thus, to try to reduce these adverse effects targeted delivery using nano-based materials could represent an alternative strategy to treat OP based on their high targeting and delivery efficiency. In fact, in this review it was found that drugs, ions, hormones, and factors, including Sr, zoledronate, and risedronate, were delivered by nano-based materials and principally through injectable and implant-based delivery strategies. The injectable delivery strategy doubtless represents a practical and minimally invasive approach, but larger defects resulting from OP often require the implantation of medical devices/biomaterials able to mimic bone. In this context, the nano-based materials displayed potential for bone tissue repair and regeneration, and they are also able to efficiently load drugs and target the diseased site. In fact, in this review the developed nano-based material drug delivery systems were found to be highly effective in stimulating bone formation and defect healing as well as bone strength in OVX and/or corticosteroid induced OP animals. However, almost all (32/33) the innovative nano-based delivery systems have been assessed by in vitro and in vivo studies, and investigations, in many cases, are still in the preliminary/early research steps. In fact, our search strategy retrieved only one non-randomized controlled trial (RCT) study on ONAS used for degenerative lumbar disease in OP patients. Thus, further investigations are mandatory for clinical translation and commercial purposes.

## 5. Conclusions and Future Perspectives

Despite the advances of nano-based material as drug delivery systems against OP over the past decade, several challenges and obstacles are still present for their clinical translation. Currently, the main limits for their application and use in the clinical *scenario* are principally due to the difficulty in reproducing manufacture, characterization, and scale-up and to an incomplete knowledge of their nanotoxicity, since NPs could cause chemical and physical impairment also to healthy cells. Additionally, the unsatisfactory drug-loading capacity (currently insufficient to reach a therapeutic level), uncontrollable release kinetics, and low delivery efficiency also limits their clinical application. Thus, to allow a faster translation of the most promising nano-based materials as drug delivery systems for OP, future research needs to focus on: (1) standardizing the synthesis and characterization of nano-based materials, while also accurately and reproducibly measuring the physical and chemical properties; (2) gaining a more complete and efficient understanding of cellular responses when cells encounter NPs; (3) elucidating the interactions between NPs and other organs to reduce nanoparticle filtration phenomena before arrival at the bone tissue; (4) gaining knowledge of the controllable multiphase drug release kinetics in order to improve the therapeutic index at the diseased site; (5) designing multifunctional NPs able to combine various therapeutic agents which would provide specific therapeutic effects, such as coordinated pharmacokinetics, as well as provide the delivery of specific drug and genes doses at the same cell subpopulation. However, to give an answer to all these clinical needs, the sharing of expertise from a multidisciplinary team of clinicians and researchers will be mandatory. We believe that, in the near future, these investigations will allow the assessment of the characteristics and selectivity of nanomaterial-based drug delivery systems, thus further extending and widening their therapeutic potential.

## Figures and Tables

**Table 1:** Preclinical in vivo studies where the injectable delivery approach was used for the delivery of drugs through nano-based materials in osteoporosis (OP) condition.

Reference	Aim	Study Design	Experimental Groups	Main Characteristics of Nanomaterial	Main Results
Cai et al., 2017 [31]	Anti-OP effect of anti-miR214 delivered by PU nanomicelles modified by the acidic peptide Asp^8^ injected via tail vein	Female C57 BL/6 OVX mice	- Asp^8^-PU (200 μL); - Asp^8^-PU-anti-miR214 (200 μL)	PU nanomicelles modified by acidic peptide Asp^8^ (~80 nm), loaded with anti-miR214	↑BMD, Tb.Th, Tb.N, BV/TV, SMI, ↓Tb.Sp, Oc.S/BS, Oc.N/BPm in Asp^8^-PU-anti-miR214 vs. Asp^8^-PU
Fouand-Elhady et al., 2020 [35]	Anti-OP effect of nHA, nCh/HA and nAg/HA delivered intravenously	Female albino Wistar OVX rats	- nHA (8 mg/kg B.wt.); - nCh/HA (8 mg/kg B.wt.); - nAg/HA (8 mg/kg B.wt.); - alendronate (1 mg/kg B.wt.)	nHA, nCh/HA and nAg/HA (25.5, 28.85 and 22.73 nm)	↓SOST, BALP, BSP, RANKL, CtsK and ↑calcification in all groups vs. OVX only
Kaur et al., 2019a [39]	Anti-OP effect of nHA and mHA particles doped with Eu oxides injected intrafemorally	Female Wistar OVX rats (DM treated)	- Eu-mHA (100 μg/kg); - Eu-nHA (25, 50, 100 μg/kg)	Eu-nHA and Eu-mHA (12.27 ± 0.08 and 25.29 ± 0.15)	↓ALP in all groups vs. OP only. ↑Ca, body and dry bone weight, volume, density, peak load, ultimate stiffness, Young’s modulus in all groups vs. OP only
Kettenberger et al., 2017 [41]	Anti-OP effect of ZOL loaded nHA integrated in a cross-linked hyaluronic acid hydrogel (nHA-ZOL-Gel) injected in the femoral condyle	Female Wistar OVX rats	- nHA-Gel; - nHA-ZOL-Gel	nHA-ZOL-Gel (~200 nm)	↑MV/TV, MR and ↓DR in nHA-ZOL-Gel vs. nHA-Gel
Khajuria et al., 2015 [42]	Anti-OP effect of ZOL-HA nanoparticles (HNLZ) injected intravenously	Female Wistar OVX rats	- Saline; - ZOL (100 μg/kg); - HA (100 μg/kg); - HNLZ (25, 50, 100 μg/kg)	HA NPs (100–130 nm) loaded with ZOL	↓BSAP, PINP, OCN, TRACP-5b, CTx, Tb.Sp, ↑BV/TV, Tb.N, Tb.Th, peak load, ultimate stiffness and strength, toughness, Young’s modulus in all groups vs. saline
Khajuria et al., 2016 [43]	Anti-OP effect of RIS/ZnHA NPs injected intravenously	Female Wistar OVX rats	- saline; - RIS (500 μg/kg); - RIS/HA (250, 500 μg/kg); - RIS/ZnHA (250, 500 μg/kg)	ZnHA NPs (14.74 and 18.08 nm) loaded with RIS	↓BSAP, TRACP-5b, Ca, P, creatinine, Tb.Sb, ↑BV/TV, Tb.N, Tb.Th, peak load, ultimate stiffness, strength, toughness, Young’s modulus, Ca, P, Ca/P in all groups vs. saline
Khajuria et al., 2017 [44]	Anti-OP effect of Sr substituted HA-ZOL (SrHA/ZOL) injected intravenously	Female Wistar OVX rats	- Saline; - ZOL (100 μg/kg); - SrHA (100 μg/kg); - SrHA/ZOL (25, 50, 100 μg/kg)	SrHA NPs (31.28–40.87 nm) loaded with ZOL	↑BV/TV, Tb.N, Tb.Th, peak load, ultimate stiffness and strength, toughness, Young’s modulus and ↓BSAP, TRACP-5b, Tb.Sp in all groups vs. saline
Kotak et al., 2020 [45]	Anti-OP effect of SCT loaded HA-NPs injected sublingually or subcutaneously	Female Sprague Dawley OVX rats	- Saline; - Subcutaneous SCT injection (200 IU daily dose); - Sublingual SCT-HA-NPs injection (200 UI daily dose)	HA-NPs (100 nm) loaded with SCT	↓serum ALP, Ca, P, erosions, porosity, resorption pits and ↑bone density and strength in all groups vs. saline
Santhosh et al., 2019 [49]	Anti-OP effect of a RIS functionalized chitosan NPs (RISCN) injected intramuscularly	Female Wistar OVX rats (MP treated)	- MP; - MP-RIS; - MP-RISCN (250 and 350)	RIS functionalize NPs	↑BMD, ALP in all groups vs. MP. ↑Ca in MP-RISCN vs. MP. ↑healing of trabecular microarchitecture and ↓cortical porosity on the bone surfaces of treatment groups
Sahana H et al., 2013 [50]	Anti-OP effect of RIS-HA NPs (NHLR) injected intravenously	Female Wistar OVX rats	- NHLR (250, 350, 500 μg/kg); - RIS (500 μg/kg)	NPs of HA (80–130 nm) loaded with RIS	↑BMD, maximum stress, Young’s modulus, ↓bone porosity in NHLR (250 μg/kg) vs. OVX only
Sun et al., 2016 [52]	Anti-OP efficacy of Ser-Asp-Ser-Ser-Asp peptide (SDSSD)-modified PU nanomicelles to deliver anti-miR-214 to OBs injected via tail vein	Female OVX mice	- SDSSD-PU-anti-scramble; - SDSSD-PU-anti-miR-214 (siRNA dose 10 mg/kg/week)	PU nanomicelles (70 nm) conjugated to SDSSD peptide to encapsulate siRNA/microRNA	↓miR-214, ↑BMD, MAR in SDSSD-PU-anti-miR-214 vs. SDSSD-PU-anti-scramble
Wang et al., 2019 [55]	Anti-OP effect of NOB-loaded PEG-PCL injected intraperitoneally	C57Bl/6 OVX mice	- NOB; - PEG-PCL; - NOB-PEG-PCL	PEG-PCL micelles loaded with NOB (diameter 124 nm)	↑BMD, BV/TV and ↓Tb.Sp in NOB-PEG-PCL vs. all groups
Zhang et al., 2014 [58]	Anti-OP effect on alveolar bone change of *N*-(2-hydroxypropyl) methacrylamide NPs loaded with Asp^8^-(STR-R8)-Sema4d siRNA injected intravenously	Female Balb/c OVX mice	- PBS; - E2; - Asp^8^-(STR-R8)-Sema4d siRNA	Polymeric NPs loaded with Asp^8^-(STR-R8)-Sema4d siRNA	↑BV/TV, OBs, OCs number, ↓Sema4d, inter-molar alveolar bone height loss in Asp^8^-(STR-R8)-Sema4d siRNA vs. all groups

Abbreviations: OP = osteoporosis; PU = polyurethane; OVX = ovariectomy; BMD = bone-mineral density; BV/TV = bone volume/total volume; Tb.Th = trabecular thickness; Tb.Sp = trabecular spacing; Tb.N = trabecular number; SMI = structure model index; Oc.S/BS = osteoclast surface/bone surface; Oc.N/BPm = Oc number/bone perimeter; HA = hydroxyapatite; nHA = nanohydroxyapatite; nCh/HA = chitosan/hydroxyapatite nanocomposites; nAg/HA = silver/hydroxyapatite nanoparticles; SOST = serum sclerostin; BALP = bone alka-line phosphatase; BSP = bone sialoprotein; CtsK = cathepsin K; Eu = europium; mHA = microsized HA; ALP = Alkaline Phosphatase; ZOL = Zoledronate; MV/TV = Mineralized volume/tissue volume; MR = Mineralization rate; DR = Demineralization rate; BSAP = bone-specific alkaline phosphatase; PINP = procollagen type I N-terminal propeptide; TRACP-5b = tartrate-resistant acid phosphatase 5b; OCN = Osteocalcin; Ca = calcium; RIS = risedronate sodium; SCT = salmon calcitonin; Sr = strontium; MP = methylprednisolone; NOB = Nobiletin; PEG = poly(ethylene glycol); PCL = polycaprolactone; OCs = osteoclasts; OBs: osteoblasts.

**Table 2:** Preclinical in vivo studies where the implant-based delivery approach was used to deliver drugs through nano-based materials in OP condition.

Reference	Aim	Study Design	Experimental Groups	Main Characteristics of Nanomaterial	Main Results
Alghamdi et al., 2014 [30]	Anti-OP effect of Ti implants coated with alendronate loaded nCaP implanted in femoral condyle	Male Wistar ORX rats	- Ti-non-coated; - Ti-nCaP; - Ti-nCaP/BP; - Ti-BP	Pin-shaped implants of pure Ti coated by ESD with nCaP, nCaP/BP, BP	↑%BV in Ti-nCaP/BP and Ti-BP vs. Ti-non-coated. ↑%BIC in Ti-nCaP and Ti-nCaP/BP vs. Ti-non-coated
Cheng et al., 2016 [32]	Anti-OP effect of NT structures loaded with Ag and Sr (NT-Ag.Sr) on Ti implants implanted in a tibial defect	Female Sprague Dawley OVX rats	- Ti; - TiO_2_-NTs; - NT_10_-Ag_1.5_Sr_3_; - NT_10_-Ag_2.0_Sr_3_; - NT_40_-Ag_1.5_Sr_3_; - NT_40_-Ag_2.0_Sr_3_	NT-Ag.Sr on Ti surfaces (30 nm, 80 nm)	↑BV/TV, Tb.N, Conn.D, BIC, BA *ratio*, ↓Tb.Sp in all groups vs. Ti and TiO_2_-NTs
Ignjatovic et al., 2013 [36]	Anti-OP effect of a paramagnetic Co-substituted HA NPs implanted in an alveolar bone defect	Female Wistar OP rats (MP and DM treated)	- HA; - HA/Co1; - HA/Co2 (mixed to autologous blood and plasma); - empty defect	Paramagnetic Co-substituted HA NPs	↑ALP in all groups vs. OP-empty defect
Luo et al., 2015 [46]	Anti-OP effect of nanosized Sr-substituted apatite/polylactide loaded with rhBMP-2 implanted intramuscularly	Female New Zealand OVX rabbits (MP treated)	- Healthy (Sr0%, Sr0.5%, Sr5%, Sr50%); - OVX (Sr0%, Sr0.5%, Sr5%, Sr50%)	Nanosized Sr-substituted apatite/polylactide loaded with rhBMP-2	↓B% in OVX-Sr0% group vs. all OVX and healthy groups. ↑B% in OVX-Sr50% group vs. OVX-Sr0% group. ↑Ap% and ↓areas with active OBs in OVX groups vs. healthy groups
Offermanns et al., 2016 [47]	Anti-OP effect of nanotopographic implants with a Sr-functionalized Ti coating (Ti-Sr-O) implanted in tibia	Female Wistar OVX rats	- Ti (uncoated); - Ti-Sr-O (2.000 nm, 1.500 nm)	Ti-Sr-O (thickness 1.500–2.000 nm)	↑BA%, BIC% in Ti-Sr-O group vs. Ti
Qayoom et al., 2020 [48]	Anti-OP effect of calcium sulfate/nHA based NC as carrier of BMP-2, ZOL, BMSCs-derived EXO, implanted in a femur defect	Sprague-Dawley OVX rats	- NC; - NC-ZOL (systemic or local); - NC-EXO-ZOL; - NC-BMP-2-ZOL	NC functionalized with BMP-2, ZOL or EXO	↑mineralization, BV/TV, Tb.N, ↓Tb.Sp in all groups vs. NC. ↑peak fracture force in NC-BMP-2-ZOL vs. all groups
Shen et al., 2016 [51]	Anti-OP effect of a HY-Aln/BMP-2 nanoparticles embedded into the Gel/Chi multilayers on Ti6Al7Nb surfaces (Ti6Al7Nb/LBL/N) implanted into femoral epiphysis	New Zealand White OVX rabbits	- Ti_6_Al_7_Nb; - Ti_6_Al_7_Nb/LBL; - Ti_6_Al_7_Nb/LBL/NP	HY-Aln NPs loaded with BMP-2a, immersed into Gel/Chi on Ti6Al7Nb	↑interfacial strength, BV/TV, Tb.Th, new bone formation in Ti6Al7Nb/LBL/NP vs. all groups
Wu et al., 2020 [56]	Anti-OP effect of a poly (*N*-isopropylacrylamide) brush modified mesoporous HA loaded with SIM (MHA-SIM-P) on femur defect	Female Wistar OVX rats	- MHA; - MHA-SIM-P	MHA-P NPs (4 nm pores) loaded with SIM	↑BV/TV, Tb.N, OPN, BSP and ↓Tb.Sp, OCs number in MHA-SIM-P vs. all groups
Yang et al., 2020 [57]	Anti-OP effect of ZOL loaded gelatin NPs integrated porous Ti scaffold implanted in a femoral defect	Female New Zealand OVX rabbits	- Ti_6_Al_4_V-ZOL-NPs (1, 10, 50, 100, 500 μmol/L)	pDA-coated porous Ti6Al4V scaffold, integrated with ZOL loaded gelatin NPs (150 nm)	↑BV/TV in Ti6Al4V-ZOL-NPs (1, 10, 50 μmol/L) vs. OVX only
Zhang et al., 2016 [59]	Anti-OP effect of HP polyplexes (PEI, PEG) loaded with miR-26a, encapsulated in PLGA MS, immobilized on NF PLLA implanted into calvaria defect	Female C57BL/6J OVX mice	- PLLA coated polyplexes; - PLLA coated HP/mi-R26a; - PLLA with immobilized PLGA (6.5, 64-K) MS loaded with polyplexes; - PLLA with immobilized PLGA (6.5, 64-K) MS loaded with HP/miR-26a	HP polyplexes (PEI, PEG) (224 nm) loaded with miRNA (miR-26a), encapsulated in PLGA MS, immobilized on NF PLLA	↑BMD, BV/TV, Ob.S/BS, Ob.N/B.Pm, MAR, BFR, OCN in cell-free PLLA with immobilized PLGA (64-K) MS loaded with HP/miR-26a vs. all groups
Zhang et al., 2017 [60]	Anti-OP effect of PIB nanogel containing MBG loaded with Sr in a critical-sized femur defect	Female OVX rats	- Empty defect; - PIB; - PIB-OBs; - PIB-Sr-MBG; - PIB-Sr-MBG-OBs	PIB nanogels loaded with Sr-MBG and OBs	↑BMD, BV/TV, mineralization, Col I in PIB-Sr-MBG-OBs vs. all groups. ↑OCs number in -PIB-OBs vs. all groups

Abbreviations: OP = osteoporosis; Ti = titanium; BP = bisphosphonate; nCaP = calcium phosphate nanoparticles; ORX = orchidectomy; %BIC = bone-to-implant contact percentage; HA = hydroxyapatite; OVX = ovariectomy; BV/TV = bone volume/total volume; Tb.N = trabecular number; NT = nanotubular; Conn.D = connective density; Co = cobalt; MP = methylprednisolone; DM = dexamethasone; Sr = strontium; B% = de novo bone formation; OBs = osteoblasts; O = oxygen; BA% = bone area; BV/TV = bone volume/total volume; Tb.Th = trabecular thickness; Tb.Sp = trabecular spacing; Tb.N = trabecular number; BV = volume fraction; ZOL = Zoledronate; NC = nanocement; BMP-2 = bone morphogenetic protein-2; Gel = gelatin; Chi = chitosan; SIM = Simvastatin; OPN = osteopontin; BSP = bone sialo protein; PLLA = poly(L-lactic acid); MSCs = Mesenchymal stem cells; Ob.S/BS = Osteoblast surface/bone surface; Ob.N/B.Pm = osteoblast number/bone perimeter; BFR = bone-formation rate; PIB = p(N-isopropylacrylamide-co-butyl methylacrylate); Col I = Type I collagen; OCs = osteoclasts; HP = hyperbranched polymer; MBG = mesoporous bioactive glass; BMD = bone-mineral density; NF = nanofibrous.

**Table 3:** Preclinical in vivo studies where oral delivery, transdermal delivery, and intranasal delivery approaches were used for the delivery of drugs through nano-based materials in OP condition.

Reference	Aim	Study Design	Experimental Groups	Main Characteristics of Nanomaterial	Main Results
Erfanian et al., 2017 [33]	Anti-OP effect of nano-sized Ca carbonate-enriched-milk and nano-sized Ca citrate-enriched-milk delivered by gavage	Female Sprague-Dawley OVX+ low-Ca diet rats	- Nano-sized Ca carbonate-enriched-milk; - Nano-sized Ca citrate-enriched-milk	Ca carbonate nano-sized particle enriched milks (~0.229–0.452 nm) and Ca citrate nano-sized particle enriched milks (~0.259–0.497 nm)	↑ Ca, Ca absorption, maximum load, femur structure morphology in nano-sized Ca carbonate-enriched-milk vs. nano-sized Ca citrate-enriched-milk
Fazil et al., 2016 [34]	Anti-OP effect of PLGA NPs and RIS delivered intranasally	Female Wistar OP rats (DM treated)	- RIS; - RIS-NPs; - RIS intravenous	PLGA-NPs loaded with RIS (184.87 ± 4.33 to 77.86 ± 8.67 nm)	↓ALP, creatinine, ALT, AST and ↑Ca in all groups vs. OP only
Jiang et al., 2015 [37]	Anti-OP effect of CIT-SO self-assembled into nanomicelles under the action of DOC administrated orally	Sprague Dawley OVX rats	- SO; - EV; - CIT (40, 20, 10 mg/kg); - CIT-SO (40, 20, 10 mg/kg)	CIT-SO-DOC nanomicelles (204.77 ± 6.81 nm and 100.80 ± 7.21 nm)	↑BMD, BMC, TMC, TMD, VOB, Tb.Th, BV/TV, Tb.N, energy to failure, stiffness, ultimate load, OPG, OCN, OPG and ↓Tb.Sp, BS/BV, CalibTbSp3D, HOP, ALP, TRACP-5b and RANKL in EV, CIT (40, 20 mg/kg) and CIT-SO (40, 20 mg/kg) vs. OVX only, and in CIT-SO vs. CIT
Kang et al., 2012 [38]	Anti-OP effect of RGD-tetrapeptide (peptide Arg-Gly-Asp-AA) modified 17β-amino-11α-hydroxyl-androst-1,4-diene-3-one nanomaterial administered orally	ICR OP mice (prednisone treated)	- Saline; - 17β-[Boc-Arg(Tos)-Gly-Asp(OBzl)-Ser(Bzl)-amido]-11α-hydroxylandrost-1,4-diene-3-one (4a); - 17β-[Boc-Arg(Tos)-Gly-Asp(OBzl)-Val-amido]-11α-hydroxylandrost-1,4-diene-3-one (4b); - 17β-[Boc-Arg(Tos)-Gly-Asp(OBzl)-Phe-amido]-11α-hydroxylandrost-1,4-diene-3-one (4c)	Pharmacophore of 17β-amino-11α-hydroxyl-androst-1,4-diene-3-one, targeting sequence of RGD-tetrapeptide (55–200, 24–182, 48–188 nm)	↑ BMD, dry weight, ash weight, Ca^2+^, BMC in all groups vs. OP-saline (4a > 4b > 4c)
Kaur et al., 2019b [40]	Anti-OP effect of transdermal NE gel loaded with LNG	Male Albino Wistar OP rats (DM treated)	- LNG5 (5 mg/kg/d); - LNG10 (10 mg/kg/d); - alendronate (0.03 mg/kg/d)	NE gel (11–123 nm) loaded with LNG	↓ALP, BALP, CTx, TRACP-5b, ↑Ca, P, OCN, PINP, Young’s modulus, peak load in LNG5 and LNG10 groups vs. OP only. ↑BV/TV, Tb.Th, Tb.N and ↓Tb.Sp in all groups vs. OP only
Takeuchi et al., 2016 [53]	Anti-OP effect of transdermal E2-loaded PLGA NPs	Female Sprague Dawley OVX + low-Ca diet rats	- PD (square gauze sheet with 12 mg of E2-loaded PLGA); - IP (two square gauze sheets with 12 mg of E2-loaded PLGA NPs at 3 V/cm)	E2-loaded PLGA NPs (165.0 ± 13.1 nm)	↑BMD in IP vs. all groups
Takeuchi et al., 2017 [54]	Anti-OP effect of a E2-loaded PLGA NPs transdermal administered using iontophoresis	Female Sprague Dawley OVX + low-Ca diet rats	- Bare NPs; - PVA-coated NPs	Bare and PVA-coated PLGA NPs (110 ± 41 nm and 106 ± 30.9 nm) loaded with E2	↑BMD in Bare NPs vs. all groups

Abbreviations: OP = osteoporosis; OVX = ovariectomy; Ca = calcium; PLGA = Poly Lactic-co-Glycolic Acid; RIS = risedronate sodium; ALP = Alkaline Phosphatase; CIT = Circinal–icaritin; SO = suet oil; EV = estradiol valerate; BMD = bone-mineral density; BV/TV = bone volume/total volume; Tb.Th = trabecular thickness; Tb.Sp = trabecular spacing; Tb.N = trabecular number; SMI = structure model index; Oc.S/BS = osteoclast surface/bone surface; Oc.N/BPm = Oc number/bone perimeter; BS/BV = bone surface over bone volume; BMC = bone mineral content; TMC = tissue mineral content; TMD = tissue mineral density; VOB = volume of bone; CalibTbTh3D = calibration of trabecular thickness-3D; CalibTbSp3D = calibration of trabecular separation-3D; HOP = hydroxyproline; TRACP-5b = tar-trate-resistant acid phosphatase 5b; LNG = lovastatin; IP = iontophoresis; hrs = hours; PVA = polyvinylalcohol; E2 = 17 β-estradiol; MP = methylprednisolone; DM = dexamethasone.

**Table 4:** Clinical studies on nano-based materials as drug delivery systems in OP condition.

Reference	Aim	Study (Trial) Type	Patient Groups	Main Characteristics of Nanomaterial, and Drug Delivery Strategy	Measurements	Main Results
Qu et al., 2017 [29]	Efficacy and safety of ONAS on DLD OP patients	Non-RCT	96 DLD OP patients (59 males, 37 females) underwent PLIFC treated with: - Control (100 mg PG); - ONAS (100 mg daily oral administration)	PG and ONAS prepared with ampicillin (200 nm)	Exp. Time: 1 month. RT-PCR (miR-155 serum levels), biochemical analysis (SOD, GSH, AST, ALT), ELISA (IL-1β, IL-1ra), clinical outcome (VAS, JOA and ODI scores, surgical duration, blood loss, abnormal motion of the surgical segment, fusion rate)	↓miR-155, ALT, AST, IL-1β, infection rates, side effects and ↑SOD, GSH, IL-1ra, fusion rates, JOA scores in ONAS group vs. control group

Abbreviations: ONAS = oligosaccharide nanomedicine of alginate sodium; DLD = degenerative lumbar disease; OP = osteoporosis; RCT = randomized controlled trial; PLIFC = posterior lumbar intervertebral fusion with cages; Exp = experimental; vs = versus; PG = pluronic nanoparticles; JOA = Japanese Orthopedic Association; RT-PCR = Reverse transcriptase-polymerase chain reaction; ODI = Oswestry Disability Index; VAS = Visual Analogue Scale; ELISA = enzyme-linked immunosorbent assay; ALT = alanine aminotransferase; AST = Aspartate aminotransferase; SOD = superoxide dismutase; GSH = glutathione; IL-1ra = interleukin-1 receptor antagonist; IL-1β = Interleukin-1 beta.

## Data Availability

No new data were created or analyzed in this study. Data sharing is not applicable to this article.
